# Prevalence and Associated Factors of Mask-Induced Acne (Maskne) in the General Population of Jeddah During the COVID-19 Pandemic

**DOI:** 10.7759/cureus.26394

**Published:** 2022-06-28

**Authors:** Rinad a Bakhsh, Shahd Y Saddeeg, Khadijah M Basaqr, Bashair M Alshammrani, Bader S Zimmo

**Affiliations:** 1 Department of Medicine and Surgery, King Abdulaziz University Faculty of Medicine, Jeddah, SAU; 2 Faculty of Medicine, Department of Dermatology, King Abdulaziz University, Jeddah, SAU; 3 Department of Dermatology, King Abdulaziz University Hospital, Jeddah, SAU

**Keywords:** jeddah, pandemic, mask wearing, covid-19, face mask, acne

## Abstract

Background: COVID-19 is a newly emerged coronavirus (SARS-CoV2) infectious disease pandemic that originated in Wuhan, China at the end of 2019. The first documented case in Saudi Arabia was on March 2, 2020. Soon after, the World Health Organization declared the outbreak a global pandemic. The ministry of health in Saudi Arabia mandated the wearing of facial masks for the general population, among other methods of protection from the spread of this highly contagious virus.

It was observed that the continuous and prolonged wearing of facial masks has led to the development of multiple skin complications and facial dermatosis, including new-onset acne as well as flares of pre-existing acne in the general population and especially healthcare workers.

Methodology: This cross-sectional study was conducted during the COVID-19 pandemic from January to September 2021 in Jeddah, Saudi Arabia. A self-administered electronic survey was distributed to residents of Jeddah city using multiple social media platforms to assess the prevalence and the factors attributed to the development of mask-induced acne.

Result: A total of 630 participants were enrolled. 470 (74.6%) were females and 160 (25.4%) were males, and the predominant age group was 18-30 years old, 374 (59.4%). The majority of participants wore a mask for five to seven days per week, 272 (43.2%), and for less than four hours per day, 378 (60%). The surgical masks were the most frequently used masks, with 597 (94.8%). Three hundred and seventy-nine (60.2%) participants had no previous skin disease on their faces. Only 251 (39.8%) of the participants had pre-existing skin diseases affecting the face. Of those, acne was the most frequent skin condition, 217 (86.4%), followed by atopic dermatitis, 12 (4.8%).

Ninety-seven (23.5%) participants without a prior history of acne reported the new onset of acne during the pandemic with the use of a face mask or face shield. Thirty people (59.9%) who had acne on their faces before the pandemic said that their acne got worse when they wore face masks or face shields for a long time during the pandemic.

Conclusion: This study demonstrated a significant association between the new onset of acne or worsening of pre-existing acne and the frequent and prolonged usage of facial masks. Thus, certain measures should be applied to prevent the development of new or worsening of prior acne while maintaining effective protection using facial masks.

## Introduction

COVID-19 is a newly emerged Coronavirus (SARS-CoV2) infectious disease pandemic that originated in Wuhan, China at the end of 2019. The first documented case in Saudi Arabia was on March 2, 2020 [1]. The World Health Organization declared it a global pandemic on March 11, 2020 [2].

The newly discovered COVID-19 virus is highly contagious and can be easily transmitted through respiratory droplets and close contact with an infected person. Therefore, the Ministry of Health in Saudi Arabia has established strict regulations to prevent the transmission of the infection and halt the spread of this pandemic. These regulations included social distancing, frequent hand washing or use of alcohol-based hand rubs, and obligatory mask-wearing in public, in addition to the use of personal protective equipment in healthcare settings [1].

Whether it is healthcare workers or individuals of the general public being exposed on daily basis, masks are being worn constantly for prolonged periods of time over many months during this pandemic [3-5]. A surge in dermatological manifestations, including new-onset acne and worsening of pre-existing acne, has been observed during the pandemic, which is likely due to frequent and prolonged mask-wearing [6,7].

Acne is a chronic inflammatory disease of the pilosebaceous unit. It is the eighth most common disease worldwide, with a prevalence rate of 9.4%. It affects 85% of people between the ages of 12 and 24, as well as 8% of adults aged 25 to 34 years old and 3% of adults aged 35 to 44 years old [8-10].

"Maskne" is a new term introduced during the COVID-19 pandemic to describe localized acne lesions confined to the facial areas covered by the mask. The areas most commonly affected are the chin, jawline, and nose, and in some cases, the forehead, where a face shield has been worn. The pathogenesis of mask-induced acne is multifactorial and represents a form of acne mechanica. Although the exact mechanism is not fully understood, mechanical pressure and friction combined with rising temperature and humidity produced by prolonged wearing of facial masks may lead to occlusive effects on the ducts of pilosebaceous units, therefore, contributing to the development of acne lesions [6,11,12].

In this study, we aim to investigate and analyse the frequency and associated factors of mask-induced acne in the general population of Jeddah, Saudi Arabia. Results can help in raising awareness, establishing risk factors, planning effective preventive measures, and optimising treatment of mask-induced acne [3].

## Materials and methods

Design/setting and ethical approval

This is a cross-sectional survey that was designed to assess the prevalence and the associated factors responsible for developing mask-induced acne in the population of Jeddah. The study was conducted during the COVID-19 pandemic from January 2021 to September 2021 in Jeddah, Saudi Arabia. It was approved by the biomedical ethical committee at King Abdulaziz University Hospital (KAUH), ref: no 121-21.

Participant selection

An anonymous, online self-administered survey questionnaire was used to collect data, which was validated by two board-certified dermatologists from King Abdulaziz University Hospital. The survey was distributed to residents of Jeddah city via numerous social media platforms (Twitter, Whatsapp, and Facebook). Participants living in Jeddah city and above 12 years old were included. Participants from other cities and responses with missing data were excluded from the study. The calculated sample size was 385 respondents with a 95% confidence level and a margin error of 5%. These calculations were performed using a Raosoft sample size calculator. The study objectives were explained, and consent was obtained at the beginning of the survey.

The survey questionnaire consisted of 20 multiple-choice questions, which were divided into four main sections: (1) demographic data including residency, age, gender, and occupation. (2) Mask wearing details including the type of mask worn, the frequency of wearing the mask per week, the duration of wearing the mask in hours per day, the frequency of changing the mask, the use of a face shield along with the masks, and the use of facial products with the mask, such as cleansers, moisturizers, and sunscreen products. (3) Underlying skin condition and skin-related adverse effects including the previous history of acne or skin diseases, development of new-onset acne or flare-ups during the pandemic. (4) Details of participants’ acne, including the type and location of acne on the face, associated symptoms (itching, dry skin, greasy skin, moisture, heat, and excess facial sweating), and methods used to alleviate acne (medical consultation, home remedies, taking off the mask periodically, moisturiser and/or cleanser use), and the effect of mask-induced acne on the participants' occupations.

Data analysis

Data entry was performed using a survey that was conducted using Google forms, and then statistical analysis was done using IBM© SPSS© Statistics version 21 (IBM© Corp., Armonk, NY, USA). Categorical variables were calculated using frequencies and percentages, the continuous variables were calculated using measures of central tendency, and chi-square was used in the analysis. P-value < 0.05 was considered statistically significant.

## Results

A total of 630 respondents were included in this study. The majority were females [470 (74.6%)]. The commonest age group was 18-30 years old, with 374 (59.4%), followed by those who were older than 30 years of age, with 162 (25.7%). Most respondents were either non-employed or non-healthcare workers [434 (68.9%) and 143 (22.7%), respectively]. Only 53 (8.4%) of the respondents were healthcare workers. Further demographics are demonstrated in Table 1.

**Table 1:** The sociodemographic characteristics of the participants (n=630).

Characteristics of participants	Frequency (%)
Gender	
Female	470 (74.6)
Male	160 (25.4)
Age groups	
Less than 18 years	94 (14.9)
18-30 years	374 (59.4)
More than 30 years	162 (25.7)
Occupation	
Healthcare worker	53 (8.4)
Non-healthcare worker	143 (22.7)
Non-employee	434 (68.9)

When asked about the number of days participants wore face masks per week, the commonest answer was five to seven days [272 (43.2%)] followed by one to two days [186 (29.5%)]. Regarding the number of hours per day participants wore face masks, 378 of the participants responded with less than four hours (60%), followed by 234 by four to eight hours (37.1%). The most frequently used mask was the surgical mask by 597 of the participants (94.8%), followed by a fabric mask by 33 (5.2%), and none used the N95 mask. Eleven (1.7%) participants used a face shield on top of their masks. The majority of participants [440 (69.8%)] reported changing their face masks every day, whereas 132 (21%) of them wore their masks for at least two to three days before changing to a new one (Table 2).

**Table 2:** The participants’ responses regarding the usage of face masks (n=630).

Average days of face mask-wearing/week	Frequency (%)
1-2 days	186 (29.5)
3-4 days	172 (27.3)
5-7 days	272 (43.2)
Average time (hours) of face mask-wearing duration/day	
<4 h/day	378 (60)
4-8 h/day	234 (37.1)
>8 h/day	18 (2.9)
The most frequent type of face mask used	
Surgical mask	597 (94.8)
Fabric face mask	33 (5.2)
Surgical mask with fabric covering	0
N95 mask	0
Frequency of mask changing	
Change mask every day	440 (69.8)
Use at least 2-3 days before changing	132 (21)
Use same mask >3 days	58 (9.2)
Wearing an additional face shield	
Yes	11 (1.7)
No	619 (98.3)

When asked if participants used any face products under the mask, 325 of the participants reported using a moisturizer (51.6%), followed 218 using makeup (34.6%). On the other hand, 263 (41.7%) of the participants did not use any products (Table 3).

**Table 3:** Frequency of face products used by participants (n=630).

Use of products on the face (multiple responses)	Frequency (%)
Moisturizer	325 (51.6)
Make-up	218 (34.6)
Topical sunscreen	147 (23.3)
None	263 (41.7)

**Figure 1:**
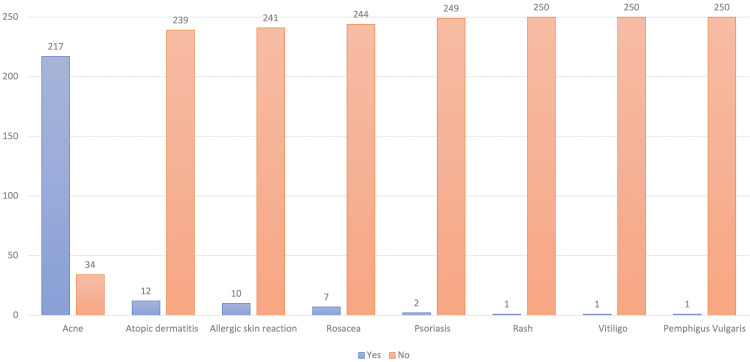
Previously diagnosed skin diseases reported by the participants (n=251).

The majority of participants, 379 (60.2%), were not diagnosed with any skin disease prior to the COVID-19 pandemic. Of the remaining participants with pre-existing skin conditions, acne was the most frequent skin condition [217 (86.4%)] followed by atopic dermatitis [12 (4.8%)], allergic skin reactions [10 (4%)], and rosacea [7 (2.8%)] (Table 4 and Figure 1).

**Table 4:** Participants diagnosed with skin conditions prior to the COVID-19 pandemic (n=630).

	Yes (%)	No (%)
History/pre-existing skin disease on face	251(39.8)	379 (60.2)
Skin disease (n=251)		
Acne	217 (86.4)	34 (13.6)
Atopic dermatitis	12 (4.8)	239 (95.2)
Allergic skin reaction	10 (4)	241 (96)
Rosacea	7 (2.8)	244 (97.2)
Psoriasis	2 (0.8)	249 (99.2)
Rash	1 (0.4)	250 (99.6)
Vitiligo	1 (0.4)	250 (99.6)
Pemphigus vulgaris	1 (0.4)	250 (99.6)

Among participants without a prior history of acne, only 97 (23.5%) participants reported the new onset of facial acne during the pandemic with continuous and prolonged use of masks. On the other hand, 130 (59.9%) of those who reported having acne before the COVID-19 pandemic answered "Yes" regarding the increase in acne severity during the pandemic (Table 5).

**Table 5:** Frequency of participants with pre-existing acne and participants who developed new-onset acne.

	Frequency (%)
Pre-existing acne (before the pandemic-related mask-wearing) (n=630)	
Yes	217 (34.4)
No	413 (65.6)
Developed new acne, after starting to wear a mask or face shield (n=413)	
Yes	97 (23.5)
No	316 (76.5)
Worsened or fared up after starting to wear a mask or face shield (n=217)	
Yes	130 (59.9)
No	87 (40.1)

Whitehead acne was the most reported type of acne experienced by 291 of the participants (92.7%), followed by papules by 76 (24.2%). When asked about the location of acne lesions on the face, the most common locations were the cheeks by 262 of the participants (83.4%), forehead by 189 (60.2%), and chin by 176 (56.1%). The nose and jawline were involved in 83 (26.4%) and 75 (23.9%) participants, respectively. Using a skin cleanser [206 (65.6%)] and a moisturizer [108 (34.4%)] were among the top methods used by participants to alleviate their acne. Only 88 (28.0%) participants answered, "I take the mask off every now and then" (Table 6).

**Table 6:** Frequencies of the types, face locations, and the means used to manage and prevent acne (n=314).

Type of acne lesions (multiple answers)	Yes (%)	No (%)
Whitehead acne	291 (92.7)	23 (7.3%)
Papules	76 (24.2)	238 (75.8%)
Blackhead acne	30 (9.6)	284 (90.4%)
Pustules	14 (4.5)	300 (95.5%)
Location of acne on face (multiple answers)		
Cheeks	262 (83.4)	52 (16.6%)
Forehead	189 (60.2)	125 (39.8%)
Chin	176 (56.1)	138(43.9%)
Nose	83 (26.4)	231 (73.6%)
Jawline	75 (23.9)	239 (76.1%)
Behind ears	8 (2.5)	306 (97.5%)
Steps adopted for prevention and/or management (multiple answers)		
Using skin cleanser	206 (65.6)	108 (34.4%)
Using moisturiser	108 (34.4)	206 (65.6%)
I take the mask off every now and then	88 (28.0)	226 (72.0%)
Self-treatment	75 (23.9)	239 (76.1%)
Consulting dermatologist	64 (20.4)	250 (79.6%)
None	62 (19.7)	252 (80.3%)

Associated symptoms reported with mask-wearing were high humidity by 167 (53.2%), oily skin by 165 (52.5%), itching by 145 (46.2%), excessive skin moisture (humidity) by 125 (39.8%), sweating by 115 (36.6%), and dry skin by 46 (14.6%) (Table 7).

**Table 7:** The common symptoms associated with wearing a mask (n=314).

Associated skin manifestations (multiple choices)	Frequency (%)
Excessive skin moisture (humidity)	167 (53.2)
Oily skin	165 (52.5)
Itch	145 (46.2)
High temperature	125 (39.8)
Sweating	115 (36.6)
Dry skin	46 (14.6)

**Figure 2:**
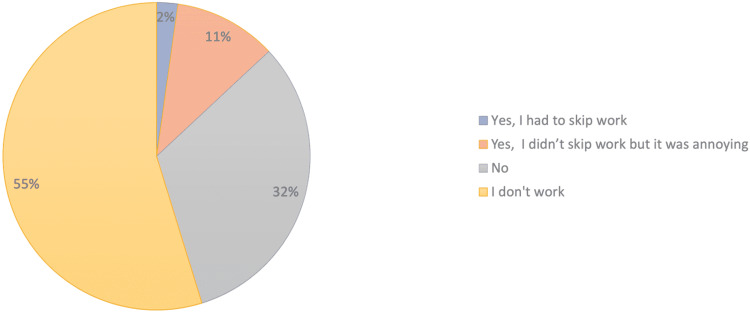
The effect of mask-induced acne on the quality of our participants' jobs (n=314).

One hundred and one (32.2%) participants denied that their mask-induced acne had affected their ability to work, while only seven (2.2%) participants had to skip work due to the unfortunate effect of acne (Figure 2).

In this analysis, the variable outcomes were the new onset of acne and increased severity of pre-existing acne, after the use of face masks and the risk factors associated with them during the COVID-19 pandemic. The results showed statistically significant associations between the type of mask used and the duration of wearing and reusing the mask. However, no significant correlations were found between using sunscreen and developing a new-onset of acne or between using makeup and flaring up acne (Tables 8-9).

**Table 8:** Correlation between the new onset of acne and risk factors (n=97). *Chi-square test was used in this analysis.

	N	%	P-value
Average time (hours) of face mask-wearing duration/day			
<4 h/day	53	54.6	0.001*
4-8 h/day	40	41.2	
>8 h/day	4	4.1	
Average days per week of face mask-wearing			
1-2 days	13	13.4	0.005*
3-4 days	31	32.0	
5-7 days	53	54.6	
The most frequent type of face mask used			
Fabric face mask	8	8.2	0.002*
Surgical mask	89	91.8	
Frequency of mask changing			
Change mask every day	71	73.2	0.004*
Use at least 2-3 days before changing	15	15.5	
Use same mask >3 days	11	11.3	
Using make-up			
Yes	50	51.5	0.005*
No	47	48.5	
Using sunscreen			
Yes	23	23.7	0.070
No	74	48.5	
Using moisturizer			
Yes	55	56.7	0.002*
No	42	43.3	

**Table 9:** Correlation between worsening or flaring up of acne and risk factors (n=130). *Chi-square test was used in this analysis.

	N	%	P-value
Average time (hours) of face mask-wearing duration/day			
<4 h/day	79	60.8	0.001*
4-8 h/day	48	36.9	
>8 h/day	3	2.3	
Average days per week of face mask-wearing			
1-2 days	41	31.5	0.004*
3-4 days	33	25.4	
5-7 days	56	43.1	
The most frequent type of face mask used			
Fabric face mask	16	12.3	0.001*
Surgical mask	114	87.7	
Frequency of mask changing			
Change mask every day	77	59.2	0.002*
Use at least 2-3 days before changing	43	33.1	
Use same mask >3 days	10	7.7	
Using make-up			
Yes	45	34.6	0.090
No	85	65.4	
Using sunscreen			
Yes	23	17.7	0.002*
No	107	82.3	
Using moisturizer			
Yes	61	46.9	0.002*
No	69	53.1	

## Discussion

The first confirmed case of the highly contagious COVID-19 infection in Saudi Arabia was on March 2, 2020. Since then, the virus has disseminated rapidly locally and worldwide [13]. Thus, regulations on social distancing and wearing masks by the general population in public areas have become mandated by many countries, including Saudi Arabia. These measures were enforced to help prevent the transmission of the infection and stop the spread of this pandemic. As a result, facial masks had to be worn frequently and for prolonged periods of time [1]. Multiple studies done in Lahore, Indonesia, Italy, and New York, among others, demonstrated an increased frequency of acne associated with mask-wearing during the COVID-19 pandemic [3,14-16]. In this study, we aimed to assess the prevalence and the factors responsible for developing mask-induced acne in the population of Jeddah, Saudi Arabia.

Females were the dominant gender in our study [470 (74.6%)]. Subjects aged between 18 and 30 years of age were the majority [374 (59.4%)]. These findings were similar to other studies in previous literature. In a study done in Indonesia by Christopher et al., females’ predominance was represented by 134 (67%) of participants with a predominant age group of fewer than 25 years, 114 (57%) [14]. Another study in Lahore by Hayat et al. showed a similar female predominance of 102 (67.66%) with a mean age of 30.5 years. This is best explained by the fact that women and people in this particular age group tend to care more about their skin and are more likely to seek medical attention for their skin health [3].

More than half of the participants wore the mask for a duration of fewer than four hours [378 (60.0%)]. This may be due to the fact that 434 (68.9%) of our participants were unemployed and 143 (22.7%) were non-healthcare workers, which can preclude the need to use the mask for prolonged hours. Nevertheless, our participants predominantly wore masks five to seven days per week [272 (43.2%)]. In contrast, a study by Özkesici in Turkey showed that 122 (88.4%) of their respondents wore masks for more than six hours, but the study included only healthcare workers, which can explain this result [17].

Surgical masks were the most commonly used type of mask, with 597 (94.8%). This is expected since surgical masks are convenient and affordable. On the other hand, a minority used fabric masks, 33 (5.2%). Similar findings were reached by Techasatian et al. in a study done in Thailand, where surgical masks represented 526 (63.15%) [18]. While in studies conducted in Lahore (Hayat et al.) and New York (Rosner), the KN95 and N95 were the most frequently used types of masks due to the fact that the mentioned studies were targeted at healthcare workers [3,16]. In our study, we found that the majority of our participants who reported new-onset acne were using surgical masks [89 (91.8%)] and only 8 (8.2%) were using fabric masks. On the other hand, 114 (87.8%) of participants who reported experiencing aggravation of the previous were surgical mask users, while 16 (12.3%) were fabric mask users. In a Lahore study, Hayat et al. found that acne, in general, was reported with the use of KN95 masks in 48 (56%) of individuals, in 25 (30%) using surgical masks, and in 12 (14%) using N95 masks [3].

On further notice, 251 (39.8%) of our participants had been diagnosed with skin diseases on their faces prior to the pandemic. Acne was the most commonly reported condition in 217 (86.4%) participants, followed by atopic dermatitis in 12 (4.8%) and allergic skin reactions in 10 (4%). In the Indonesia study, Christopher et al. reported that 48 (36.1%) of respondents had a prior skin disease; the most common were: dermatitis (29.3%), acne (3.0%), urticaria (1.5%), and psoriasis (1.5%) [14].

In the current study, 217 (86.4%) of our sample had acne before the emergence of COVID. We found that there was an increase in the percentage of acne onset and flare-ups with the necessary regular use of a mask during the pandemic. Ninety-seven (23.5%) participants had new-onset acne, while 130 (59.9%) experienced worsening of their previous acne. These findings were comparable to other studies in the literature. Hayat et al. found an increased percentage of new acne in 61 (72%) of participants and acne exacerbation in 24 (28%) [3].

Cheeks were the most frequent acne location on the faces of 262 (83.4%), while in the study by Hayat et al., the chin was the most involved area in 73 (86.5%) of the participants. White comedonal lesions [291 (92.7%)] were far more frequent than inflammatory (papulo-pustular) lesions and black comedones. This contrasts with the findings by Hayat et al. where inflammatory (papulo-pustular) lesions were predominant in 58 (68%) of participants [3]. In addition, excessive skin moisture (humidity), oily skin, and itching were the most recurring reported symptoms under the mask among our sample, in contrast to the study by Christopher et al., in which dryness/tenderness, itch, and rash were the most reported symptoms. The hot and humid climate of Jeddah city can be a contributing factor to our findings [14].

Our research has investigated the possible factors that may contribute to the new onset of acne and the correlation was found to be significant, with an average number of days of wearing masks per week (p-value=0.005) and an average number of hours of wearing masks per day (p-value=0.001). The same factors also showed a significant correlation with the worsening of pre-existing acne, with a p-value=0.004 for the average number of days of wearing masks per week and a p-value=0.001 for the average number of hours of wearing masks per day. It was found that wearing the mask for more than five to seven days per week increased the likelihood of developing a new onset of acne. We can conclude that the longer the duration of wearing a mask, the greater the likelihood of developing a new acne breakout. In their research, Techasatian et al. showed that wearing a face mask for four to eight hours per day and more than eight hours per day enhanced the risk of adverse skin reactions on the face compared to wearing a face mask for less than four hours per day. Taking a short break from wearing a mask every few hours is recommended to prevent mask-related adverse skin reactions, including acne [18]. Another significant correlation was found between the frequency of reusing the same mask and acne. Participants who used the same mask multiple times were more likely to develop new acne (p-value=0.004) or flare-up their previous acne (p-value=0.002). Reusing the same mask multiple times without changing it may lead to an accumulation of residues and increase the risk of blockage of sebaceous glands, which therefore contributes to the pathogenesis of mechanical acne [18].

A positive correlation was found between the use of make-up products and the emergence of acne (p-value=0.005) as seen in 50 (51.5%) participants using makeup products. The use of makeup products can contribute to developing acne cosmetica due to the interaction of the sebum produced by the sebaceous glands and the comedogenic ingredients in these products, which can result in clogged pores [19]. Furthermore, acne flare-ups were less likely in participants who used sunscreen and moisturizers, with only 23 (17.7%) of participants using sunscreen and 61 (46.9%) of participants using moisturizers reporting increased severity of acne. However, 55 (56.7%) of respondents who used a moisturizer documented a new onset of acne, while only 23 (23.7%) of those applying topical sunscreen reported a new emergence of acne. Sunscreens have soothing and hydrating components, like moisturizers. However, the effect of sunscreens and moisturizers can be difficult to assess due to the variable comedogenicity of different topical products as well as different individual susceptibilities for acne development. Using non-comedogenic oil-free moisturizers and sunscreens remains an important part of acne treatment [20].

Facial cleansers, moisturizers, and self-treatment were what most of our participants used in an attempt to alleviate their facial acne. In general, skin care approaches and self-treatment were more frequent than seeking a dermatology consultation. This might have been due to the difficulty of seeking medical attention during periods of lockdown and fear of exposure to positive COVID-19 cases. Participants from a study conducted in New York City by Rosner also reported using lotions and creams to relieve skin breakdown [16].

Contrary to other studies, our research was directed at the general population rather than being focused on healthcare workers [3,14,16]. In addition, our study specifically focused on mask-induced acne, unlike other studies that investigated multiple adverse skin reactions [14,16,18].

Wearing masks is an important part of protective measures that are essential to prevent the spread of the COVID-19 virus. Therefore, general recommendations are advisable to prevent cutaneous adverse reactions, especially acne, while wearing the mask regularly. A skincare routine consists of a gentle facial cleanser, a light facial moisturizer, and a non-comedogenic broad-spectrum sunscreen to reduce irritation and friction and prevent acne; avoiding makeup and other comedogenic products while wearing masks; taking a break from wearing masks every four hours, whenever it is safe to do so; wearing a new surgical mask every day to avoid prolonged reuse of the same mask. In the case of fabric reusable masks, wash them regularly to remove any irritating or blocking residue [3].

Limitations

Certain limitations should be acknowledged. First, this paper did not investigate the general health conditions of participants, such as hormonal, dietary, or genetic factors or medications that can affect the results of an acne outbreak. Second, our study only targeted the Jeddah city population. Hence, our findings cannot be generalized to the whole population of the kingdom of Saudi Arabia. This is attributable to the fact that Jeddah is a seaside city, which is known for its hot and humid weather compared to other regions of the country.

## Conclusions

In the final analysis, our study results showed a significant association between the frequent usage of face masks and the emergence of a new onset of acne and the flaring up of the previous acne. Also, it was found that certain associated factors measured can lead to the new onset of acne and worsening of the previous acne. Consequently, certain recommendations should be applied routinely to prevent the development of new or worsening of pre-existing acne.
